# An alternative treatment of hyperlipidemia with red yeast rice: a case report

**DOI:** 10.1186/1752-1947-4-4

**Published:** 2010-01-08

**Authors:** James S Lin

**Affiliations:** 1 Geriatric Research Education and Clinical Center, Atlanta Veterans Affairs Medical Center, Clairmont Road, Decatur Georgia 30033, USA

## Abstract

**Introduction:**

Hyperlipidemia is prevalent and is highly associated with coronary heart disease. Some patients are reluctant or opt not to take lipid-lowering prescription medications for fear of adverse drug reactions. There are currently few well-designed randomized controlled trials showing the possibility of reducing cholesterol using red yeast rice. Meanwhile, adverse effects have also been reported.

**Case presentation:**

A 64-year-old Asian man was diagnosed with hyperlipidemia despite a healthy lifestyle. In addition to diet changes, the patient used red yeast rice and succeeded in lowering his level of serum lipids.

**Conclusion:**

Based on this case and a review of current literature, in addition to therapeutic lifestyle change, red yeast rice may be a useful alternative treatment for primary hyperlipidemia in patients with low cardiac risk and who refuse to take any lipid-lowering prescription medication or who maybe be statin intolerant.

However, primary care physicians must be aware of the potential side effects of taking red yeast rice.

## Introduction

Hyperlipidemia is highly prevalent. It is closely related to coronary heart disease which is the most common cause of death in the United States [1]. Approximately 52 million adults require lifestyle modifications including dietary changes and exercises, and 13 million adults need lipid-lowering medications to control their low-density lipoprotein (LDL) levels [2].

Primary care physicians are faced with many challenges in treating hyperlipidemia. Lifestyle modification alone is often unsuccessful in decreasing low-density lipoprotein (LDL) levels. In addition, patients who require lipid-lowering agents are oftentimes non-compliant, which is partially due to their fear of any adverse effects. It has been shown that roughly 50% of patients taking lipid-lowering drugs discontinue their medication after one year, and 75% stop after two years [3].

The growing mistrust of the general public on the pharmaceutical industry also contributes to the decision of patients not to take prescription lipid-lowering drugs. As a result, patients seek alternative drugs or opt to rely on natural therapy in order to control their hypercholesterolemia.

This case highlights a possible alternative treatment for hyperlipidemia for patients who are unwilling to take prescription lipid-lowering drugs but who are at a minimal risk of developing coronary artery diseases.

## Case presentation

A 64-year-old Asian man who was previously healthy and had no comorbidity presented for his annual physical examination. He was not taking any medications except for his daily multivitamins. He also indicated on many occasions that he would not take any prescription medications unless it was for treating a life-threatening condition.

His history and physical examination were unremarkable. His family history only included diabetes mellitus type 2 on his maternal side. He ate a balanced diet, exercised regularly, and actively got involved his community. He did not smoke and only drank a glass of red wine once a week. His blood pressure was normotensive, his body mass index (BMI) was normal, and all preventive measures he underwent were up-to-date. A routine blood work was done on that visit to identify the patient's comprehensive blood count (CBC), comprehensive metabolic panel (CMP), lipid panel, thyroid function test, and prostate-specific antigen (PSA).

The patient was phoned and informed of his blood work results three days later. His blood work was unremarkable except for the lipid panel which showed a total cholesterol level of 260, a low-density lipoprotein (LDL) level of 202, a high-density lipoprotein (HDL) level of 40, and a triglyceride level of 179. Based on his calculated 10-year total coronary heart disease risk, he was recommended to continue his regular exercises and make further dietary changes to induce more omega-3 and to cut down on total fat intake. He was also advised to return to the clinic after three months to repeat the lipid panel test.

The patient returned to the clinic three months later for his follow-up evaluation for hyperlipidemia. His vital signs and the results of his physical examination were unchanged from his previous presentation. However, he stated that in addition to dietary changes, he had also begun taking an over-the-counter dietary supplement of 600 mg of red yeast rice(brand name: Schiff) twice daily with meals for about 12 weeks after he learned that his cholesterol levels were high. He used it because he thought it was more "natural". A repeated lipid panel test surprisingly showed a reduction in his total cholesterol level (198), LDL level (155), and triglyceride level (146). Meanwhile, his HDL level was unchanged.

This patient returned to the clinic one more time three months later for his reevaluation for hyperlipidemia. Again, his vital signs and the results of his physical examination were unremarkable. While continuing on a regular exercise regimen of moderate intensity, a healthier diet, and the red yeast rice supplement, his lipid panel remained stable and satisfactory. His total cholesterol was found to be 190, LDL level was 152, triglyceride level was 142, and high- HDL level was 45.

## Discussion

Red yeast rice, a substance made by fermenting a type of red yeast called *Monascus purpureus*, has been used for centuries in China as a type of seasoning. It has also been used in traditional Chinese medicine as a remedy to improve circulation and alleviate indigestion and diarrhea [4]. In recent years, it has been established by scientists as a potentially useful product that aids in lower serum lipids, including cholesterol and triglyceride.

Researchers found that red yeast rice contains naturally-occurring monacolin, an active ingredient in the popular statin drug, lovastatin. Lovastatin inhibits HMG-CoA reductase, an enzyme that is important in synthesizing cholesterol in the body [4].

At present only a few well-designed randomized controlled trials using red yeast rice as a cholesterol lowering agent have been designed. One recent study demonstrated that red yeast rice and lifestyle modification can decrease LDL levels without increasing creatinine phosphokinase (CPK) or pain levels. It was also noted as a viable treatment alternative for patients with hypercholesterolemia who cannot tolerate statin therapy. The authors also pointed out that red yeast rice supplement used by the patients in their study contained naturally occurring lovastatin that is equivalent to about a 6 mg of daily dose. Because this is such a small dose, it was postulated that there might be something other than monacolin that may inhibit HMG-CoA reductase in red yeast rice [5]. One study also showed that lifestyle modifications coupled with the ingestion of red yeast rice and fish oil were as good in lowering LDL as taking simvastatin for 12 weeks [6]. A meta-analysis of randomized controlled trials indicated that the effects of red yeast rice in lowering total cholesterol, triglyceride and LDL levels were more significant than placebo but similar to the effects of pravastatin, simvastatin, lovastatin, atorvastatin, or fluvastatin [7].

However, just because red yeast rice is natural and viewed as a dietary supplement does not mean that it is safe and without any adverse effects. A case of acute hepatitis was reported after a patient took an over-the-counter lipid-lowering product containing red yeast rice and the liver function test returned to normal only after the supplement was discontinued [8,9]. Another commonly reported adverse effect of red yeast rice is myopathy [10,11].

## Conclusion

Based on this case and a review of current literature, it would be advisable for primary care physicians to prescribe therapeutic lifestyle change and to ask patients to consider using red yeast rice as a useful alternative treatment for primary hyperlipidemia in patients with low cardiac risk and who refuse to take any lipid-lowering prescription medication or who maybe be statin intolerant. It must be realized, however, that the cholesterol improvement seen in this case could be multifactorial, and that the potential of lifestyle modification alone should not be undermine. It must also be remembered that some of the potential adverse effects of statins, including acute hepatitis and myopathies, can also occur in patients using red yeast rice. Therefore, it would be helpful to discuss this with patients prior to starting them on red yeast rice treatment. As with statin prescription, precautions such as checking the patient's liver and renal functions should also be observed. It should also be noted that just like statins, pregnant or nursing women should avoid ingesting red yeast rice.

## Consent

Written informed consent was obtained from the patient for publication of this case report. A copy of the written consent is available for review by the Editor-in-Chief of this journal.

## Competing interests

The author declares that they have no competing interests.

## References

[B1] American Heart Association Heart and Stroke Statistical Update 1997 Dallas: American Heart Association

[B2] Sempos CT Cleeman JI Carroll MD Johnson CL Bachorik PS Gordon DJ Burt VL Briefel RR Brown CD Lippel K Rifkind BM Prevalence of high blood cholesterol among US adults: an update based on guidelines from the second report of the National Cholesterol Education Program Adult Treatment Panel JAMA 1993 269 3009 3014 10.1001/jama.269.23.3009 8501843

[B3] Roberts WC The underused miracle drugs: the statin drugs are to atherosclerosis what penicillin was to infectious disease Am J Cardiol 1996 78 377 378 10.1016/S0002-9149(96)00441-9 8759827

[B4] Red Yeast Rice http://altmedicine.about.com/od/herbsupplementguide/a/redyeastrice.htm

[B5] Becker DJ Gordon RY Halbert SC French B Morris PB Rader DJ Red yeast rice for dyslipidemia in statin-intolerant patients: a randomized trial Ann Intern Med 2009 150 830 839 19528562 10.7326/0003-4819-150-12-200906160-00006

[B6] Becker DJ Gordon RY Morris PB Yorko J Gordon YJ Li M Iqbal N Simvastatin vs. therapeutic lifestyle changes and supplements: randomized primary prevention trial Mayo Clin Proc 2008 83 7 758 764 10.4065/83.7.758 18613992

[B7] Liu J Zhang J Shi Y Grimsgaard S Alraek T Fonnebo V Chinese red yeast rice (Monascus purpureus) for primary hyperlipidemia: a meta-analysis of randomized controlled trials Chin Med 2006 1 4 10.1186/1749-8546-1-4 17302963 PMC1761143

[B8] Grieco A Miele L Pompili M Biolato M Vecchio FM Grattagliano I Gasbarrini G Acute hepatitis caused by a natural lipid-lowering product: when "alternative" medicine is no "alternative" at all J Hepatol 2009 50 6 1273 1277 10.1016/j.jhep.2009.02.021 19398239

[B9] Roselle H Ekatan A Tzeng J Sapienza M Kocher J Symptomatic hepatitis associated with the use of herbal red yeast rice Ann Intern Med 2008 149 7 516 517 18838736 10.7326/0003-4819-149-7-200810070-00021

[B10] Lapi F Gallo E Bernasconi S Vietri M Menniti-Ippolito F Raschetti R Gori L Firenzuoli F Mugelli A Vannacci A Myopathies associated with red yeast rice and liquorice: spontaneous reports from the Italian Surveillance System of Natural Health Products Br J Clin Pharmacol 2008 66 4 572 574 10.1111/j.1365-2125.2008.03224.x 18637891 PMC2561108

[B11] Mueller PS Symptomatic myopathy due to red yeast rice Ann Inern Med 2006 145 6 474 475 10.7326/0003-4819-145-6-200609190-00021 16983142

